# Therapeutic potential of natural coumarins in autoimmune diseases with underlying mechanisms

**DOI:** 10.3389/fimmu.2024.1432846

**Published:** 2024-10-31

**Authors:** Yan Li, Guan-qing Wang, Yan-bin Li

**Affiliations:** ^1^ Department of Neurology, The First Affiliated Hospital of Shandong First Medical University & Shandong Provincial Qianfoshan Hospital, Shandong Institute of Neuroimmunology, Shandong Provincial Key Medical and Health Laboratory of Neuroimmunology, Jinan, China; ^2^ College of First Clinical Medicine, Shandong University of Traditional Chinese Medicine, Jinan, China

**Keywords:** coumarins, autoimmune diseases, anti-inflammatory, MAPKs, NF-κB, epigenetic modulation

## Abstract

Autoimmune diseases encompass a wide range of disorders characterized by disturbed immunoregulation leading to the development of specific autoantibodies, which cause inflammation and multiple organ involvement. However, its pathogenesis remains unelucidated. Furthermore, the cumulative medical and economic burden of autoimmune diseases is on the rise, making these diseases a ubiquitous global phenomenon that is predicted to further increase in the coming decades. Coumarins, a class of aromatic natural products with benzene and alpha-pyrone as their basic structures, has good therapeutic effects on autoimmune diseases. In this review, we systematically highlighted the latest evidence on coumarins and autoimmune diseases data from clinical and animal studies. Coumarin acts on immune cells and cytokines and plays a role in the treatment of autoimmune diseases by regulating NF-κB, Keap1/Nrf2, MAPKs, JAK/STAT, Wnt/β-catenin, PI3K/AKT, Notch and TGF-β/Smad signaling pathways. This systematic review will provide insight into the interaction of coumarin and autoimmune diseases, and will lay a groundwork for the development of new drugs for autoimmune diseases.

## 1 Introduction

Autoimmune diseases (AIDs) are inflammatory disorders caused by immune dysfunction and loss of immune tolerance, leading to the recognition of self-antigens by the body’s immune system (1, 2). Currently, more than 80 AIDs have been identified, including rheumatoid arthritis, type 1 diabetes mellitus, and psoriasis (3). AIDs can occur at any age and are particularly more prevalent in women than in men. It is estimated that 8% to 10% of population worldwide is afflicted by AIDs (4). Autoimmunity and autoimmune diseases have been increasing dramatically in many parts of the world in recent years, possibly due to changes in our exposure to environmental factors. Current evidence suggests that major changes in our food, exogenous substances, air pollution, infections, personal lifestyles, stress, and climate change are responsible for these increases (5). Autoimmune diseases have a devastating impact on individuals and caregivers in our society, and a large amount of healthcare utilization leads to high public and private costs, and current projections suggest that they will become more prominent diseases in the future (6). In particular, AIDs pose a major challenge to the public health system, which is second only to cancer and cardiovascular diseases, due to their long cycle and susceptibility to relapse (7–10). Therefore, it is of great importance and urgency to find effective methods for the prevention and treatment of AIDs. Coumarins are a class of aromatic natural products with benzene and alpha-pyrone as its basic structure, which are widely found in Umbelliferae, Brassicaceae, Asteraceae, Leguminosae, Orchidaceae (11). Coumarins can be divided into simple coumarins, furanocoumarins, pyranocoumarins and others based on the chemical structures (12). Accumulating studies have shown that coumarins possess a variety of pharmacological activities such as anti-tumor, anti-inflammatory, and anti-osteoporosis (13). Nowadays, coumarins have been gaining more attention from investigators due to its excellent biological activities in AIDs. Here, we review the latest research data on coumarins for the treatment of AIDs with the aim of understanding the pharmacological mechanisms of coumarins and developing novel agents for the treatment of AIDs.

## 2 Pathophysiology of autoimmune diseases

The mechanism by which the immune system prevents pathogens from attacking the organism is very complex. it can remove senescent cells and immune complexes from the body through various immune cells (such as macrophages, dendritic cells (DCs), T-lymphocytes, B-lymphocytes, etc.), and at the same time, it can recognize its own tissues and cells as its “self”, thus forming immune tolerance. Immune tolerance is defined as a state in which immunologically active cells are unable to produce specific immune effector cells and specific antibodies when exposed to antigenic substances, thus failing to execute a normal immune response (9, 14). In some cases, autoimmune tolerance is disrupted and the absence of immune tolerance induces the immune system to produce autoantibodies in response to self-antigens. Antigen presenting cells (APCs) present autoantigens to T cells with the participation of major histocompatibility complex (MHC) molecules (15). T helper cells are stimulated by MHC II and release different cytokines that can directly trigger macrophages (MP), monocytes, and B cells (16). T cells control the immune response by influencing the mixture of interleukins produced. B cells produce antibodies against their own molecules that react with accessible cells and directly or indirectly mediate damage (17). When the immune system produces a strong and sustained immune response against its own tissues and cells, leading to cellular destruction or tissue damage and clinical symptoms, it can lead to AIDs (18). Broadly speaking, AIDs are diseases caused by the immune response of the immune system against its own components. All diseases caused by dysfunctions of the autoimmune system can be referred to as AIDs.

Genetic, epigenetic, and environmental factors (hormones, nutrition, drugs, microbiota, apoptosis, and others) are predisposing factors for autoimmunity (19). Although AIDs are considered rare, epidemiologic data show that nearly 3-5% of the population suffers from type 1 diabetes (T1D) and autoimmune thyroid diseases (20). According to clinical manifestations, AIDs can be categorized into two categories: systemic AIDs and organ-specific AIDs (3, 21). Systemic AIDs are those in which immune response causes pathological damage to multiple organs and tissues throughout the body, mainly including rheumatoid arthritis (RA), systemic lupus erythematosus (SLE), Sjögren′s syndrome (SS) and others. Organ-specific AIDs refer to patients whose lesions are generally confined to a specific organ and caused by an autoimmune response against the particular organ. It mainly includes Hashimoto’s thyroiditis (HT), Graves’ Disease (GD), myasthenia gravis (MG) and others.

The imbalance of immune cells activation and regulation caused by the failure of lymphocyte self-tolerance mechanisms is considered to be a major driver of the progression of human AIDs (22). The production of autoantibodies is a key event in the development of AIDs. Under the influence of T cells or innate triggers, self-tolerance is first interrupted, and the B-cell response leads to systemic autoimmunity and the production of pathogenic autoantibodies, which are the main immune abnormality in AIDs (23). Expansion of self-reactive T cells is a biomarker of many AIDs, which is essential in the orchestration of innate and adaptive immune responses and in the induction of tissue damage. Among them, CD4+ T cells make important contributions by secreting various cytokines, chemokines and cell-cell interactions. IL-17-producing CD4+ T cells (Th 17 cells) are the core of the disease pathogenesis. When activated by antigen presenting cells (APCs), CD4+ T cells differentiate into different cell lines with unique functions, including helper T (Th) 1, Th2, Th17, and regulatory T (Treg) cells, each of which secretes its own set of cytokines (24). A balance is required to maintained between Th cell activation and Treg cells-mediated inhibition to maintain effective immune homeostasis. Disruption of this balance lead to lymphocytes generating an immune response and/or producing antibodies against their own cells and tissues (25).

The T cell subsets involved in the inflammatory response are mainly Th1 and Th17. Th1 cells are generated from CD4+ T lymphocytes activated by interleukin (IL)-12 through the STAT4 signaling pathway and the transcription factor T-bet, mainly secreting cytokines, such as IL-2, IL-12, interferon-gamma (IFN-γ) and tumor necrosis factor-alpha (TNF-α), and participate in the cellular immune response (26). Th2 cells are induced by IL-4 through the STAT6 signaling pathway and the transcription factor GATA-3, mainly secreting IL-4, IL-5, IL-6, IL-10 and IL-21 to participate in the immune response. Th1 plays a certain role in inhibiting the activation of Th2, and the two regulate and constrain each other, putting the body in a dynamic balance of cellular immunity and humoral immunity (27). Th 17 cells are one of the most predominant pathogenic cells among Th cells, and their main function is to secrete cytokines IL-17A, IL-17F, and IL-22. Their activation and proliferation require multiple transcription factors (such as NF-κB, STAT3) and specific cytokines (such as transforming growth factor-β (TGF-β), IL-6, IL-23) (28, 29). Treg cells, as an important factor in the maintenance of immune tolerance by the organism, can regulate the stable state of lymphocytes. Under the induction of the specific transcriptional regulator Forkhead box protein P3 (Foxp3), they exert anti-inflammatory effects by releasing anti-inflammatory cytokines such as IL-10 and TGF-β (30).

Recent studies have confirmed the role of immune cells and cytokines in AIDs. For example, multiple sclerosis (MS) is a chronic inflammatory autoimmune disease of the central nervous system, characterized by a positive correlation between the imbalance of the Th17/Treg ratio and the severity of MS symptoms (31, 32). Similarly, inflammatory bowel disease (IBD) and SLE are AIDs characterized by elevated levels of pro-inflammatory cytokines IL-1 and LTB4 (33). Furthermore, it is known that psoriasis is caused by activation of the IL-23/Th17 cytokine axis (34, 35). In UC patients, the increasement of IL-1, IL-6 and TNF-α are observed. In addition, elevated levels of IL-6 can also be observed in patients with T1D, RA and psoriasis (36). Studies have confirmed that Th17 cells are critical for the severity of collagen-induced arthritis and RA (37). Similarly, IL-17 and TNF-α-induced increase in intestinal barrier permeability can promote the development of Crohn’s disease (CD), ulcerative colitis (UC), and MS (38, 39).

AIDs are the result of a combination of genetic predisposition and environmental factors. Genome-wide association studies (GWASs) has been widely used to identify susceptibility genes for AIDs and has identified many relevant mutations in T cells, including Single Nucleotide Polymorphism (SNPs) in IL-23R, IL-17A/F, IL-21, JAK2, STAT2, CARD9, CCR6, and others (40). In addition, epigenetic mechanisms influence the development of many AIDs under the influence of environmental factors. M6A-modified regulatory factors can be involved in T cell-mediated autoimmune diseases. It was found that the m6A-modifying demethylase ALK-BH5 promotes IFN-γ and CXCL2 mRNA stability in CD4+ T cells, which in turn enhances CD4+ T cell pathogenicity in experimental autoimmune encephalomyelitis (EAE), whereas the demethylase FTO does not function (41). In experimental autoimmune uveitis, the presence of METTL3 in autoreactive Th17 attenuates Th17 pathogenicity by enhancing ASH1L mRNA stability to reduce IL-17 and IL-23 receptor expression (42). However, in psoriasis, T cell-specific deletion of ALKBH5 instead exacerbates skin inflammation (43). Epigenetic modifications regulate the body’s inflammatory response and immune response at multiple levels through DNA methylation, histone acetylation, and microRNAs, while the DNA sequence remains unchanged (44). For example, due to the reduced expression of H3K4 methyltransferase Ash1L, Tregs in RA patients express low levels of Foxp3 while Ash1L can enhances TGF-β/Smad signaling promotes Treg differentiation, inhibits histone deacetylase 1 (HDAC1), and reduces histone deacetylation of Foxp3 (45).

## 3 Clinical status of coumarins in autoimmune disorders

**Figure 1:**
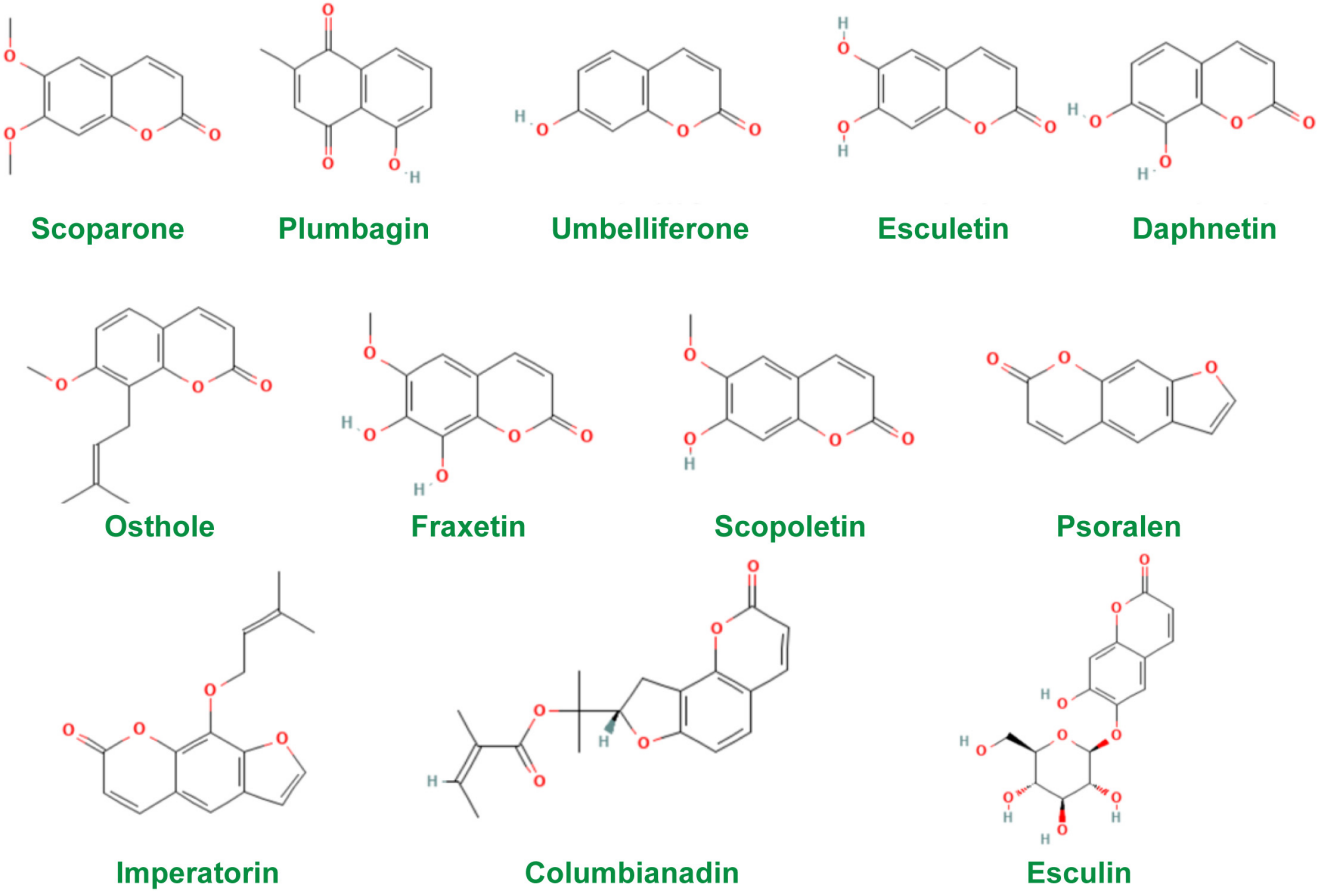
Chemical structures of coumarins used in studies.

Coumarins are a class of natural compounds widely found in nature (46). Modern pharmacological and clinical studies have shown that coumarins have pharmacological effects such as anti-tumor (47), anti-inflammatory (48), anti-osteoporosis (49), cardiovascular and neuroprotection (50), anti-bacterial (51), anti-tuberculosis (52), and photosensitization (53). In addition, coumarins are effective in the treatment of several AIDs in studies. Therefore, coumarins need more attention. The chemical structures of the constituents were screened using the PubChem database (http://pubchem.ncbi.nlm.nih.gov) and the structures of the most widely studied coumarins are given in **Figure 1**.

Given its immunomodulatory activity, coumarin has become a pharmacological tool for the treatment of various AIDs. Currently, coumarin combinations are used in the treatment of skin and AIDs such as psoriasis (54). Furanocoumarins are a class of natural plant photosensitizers. Studies have confirmed that furanocoumarins can increase the body’s sensitivity to long-wave ultraviolet light (53). PUVA, a combination of psoralen (P) and ultraviolet A (UVA), is increasingly being used to treat chronic plaque-type psoriasis and chronic palmoplantar psoriasis, and has become a second-line therapy for patients with moderate to severe psoriasis (55–61). In the treatment of severe chronic atopic dermatitis, PUVA therapy provides better short- and long-term efficacy than UV therapy alone (62–64). PUVA is also safe and effective in the treatment of cutaneous T-cell lymphoma (65). And, PUVA therapyis effective in patients with cutaneous T-cell lymphoma (CTCL) complicated by ankylosing spondylitis (AS) (66). In patients with alopecia areata (AA), dilutions of psoralen were applied to the patient’s scalp, and hair regrowth was observed in 6 of 9 patients after up to 10 weeks of treatment (67). Also, PUVA is effective in patients with AA (68, 69). In clinical practice, coumarins have been used regularly in the treatment of vitiligo. Psoralen and bergapten can increase the tolerance of human skin to radiation and produce hyperpigmentation when exposed to ultraviolet light (70). In addition, in a clinical study evaluating the photochemotherapeutic properties of bergapten microcrystalline formulations, the data results showed that bergapten was almost completely free of phototoxic and drug intolerance reactions, and that other side effects, such as severe erythema, itching, and nausea, were seen only rarely. Bergapten may be used as a photochemical therapy (PUVA) as an important alternative therapy (71). A study investigated the distribution of bergapten in the skin following oral administration of the drug. Bergapten concentrations in the skin following single and multiple oral doses of the drug were measured at healthy and psoriatic sites in 10 patients with psoriasis. The results showed that after oral administration of bergapten, accumulation levels were higher in the more external layers of the skin, the drug had a high affinity for the stratum corneum, and drug concentrations were similar in healthy and psoriasis sites, suggesting that lesions did not affect the distribution of the drug in the skin (72).

## 4 Coumarins act on immune cells and cytokines in autoimmune disorders

**Figure 2:**
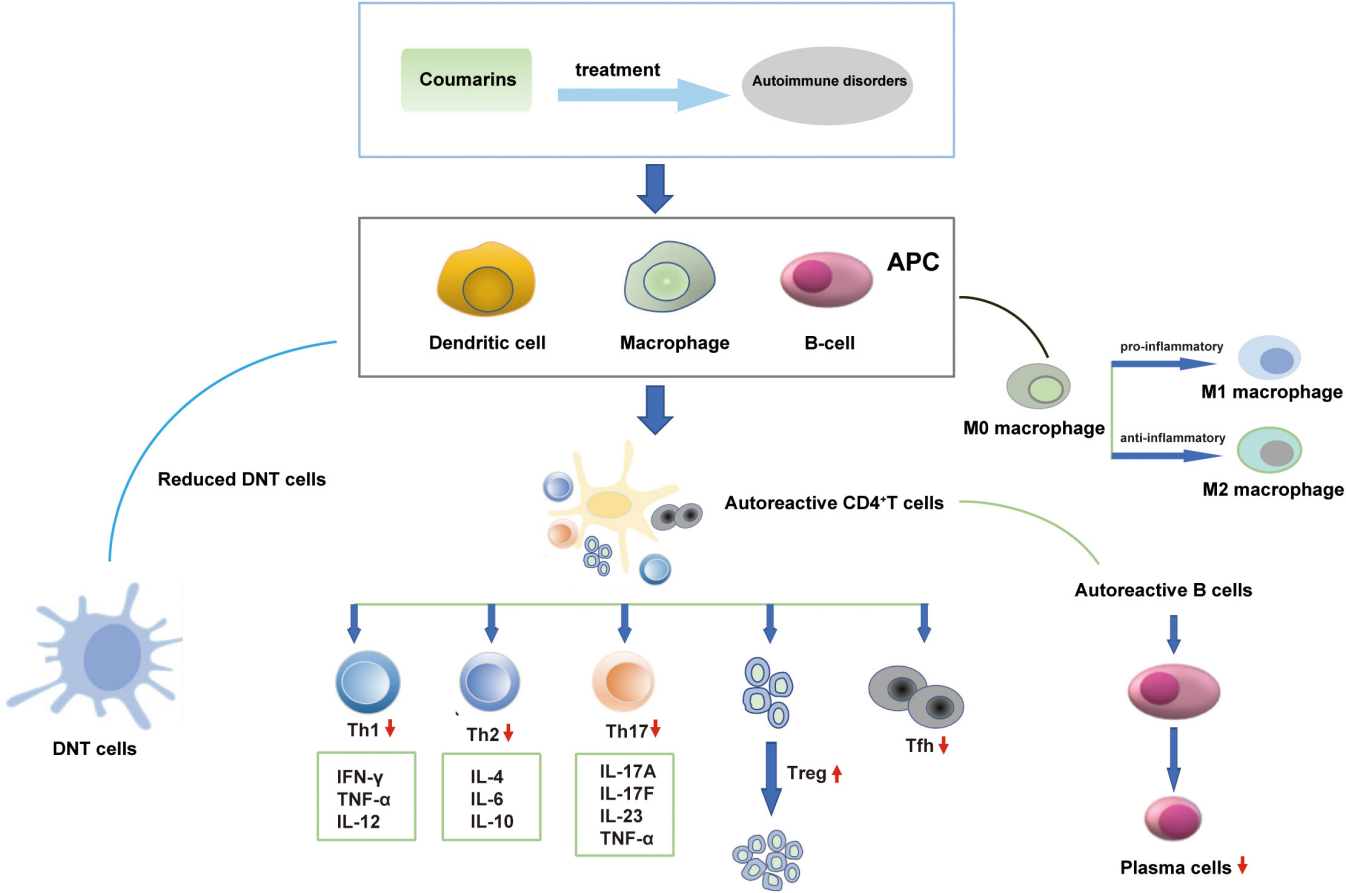
Coumarins act on immune cells and cytokines in autoimmune disorders.

AIDs have been categorized into several types and more than 80 such diseases have been identified. Coumarins can act on immune cells and cytokines to exert beneficial effects on AIDs. The regulatory effects of coumarins on significant immune cells and cytokines are shown in **Figure 2**.

RA is a chronic, systemic autoimmune disease with symmetrical, erosive, inflammation occurring in multiple joints as its main clinical manifestation. CD83 is a dendritic cell marker that belongs to the immunoglobulin superfamily and is closely associated with autoimmune diseases (73). TNF-β, which mediates a variety of inflammatory and immunostimulatory responses (74). Sterol regulatory element‐binding protein 1 (SREBP1), a transcription factor, is a major regulator of genes that control cellular lipid homeostasis (75). Synergistic treatment with Imperatorin and β‐sitosterol significantly up-regulated the expression levels of TNF-β, CD83, and SREBP1 in peripheral blood CD4+ T cells, which improved the severity of arthritis in collagen-induced arthritis (CIA) rats (76). In addition, imperatorin inhibits cell proliferation and induces apoptosis in RA fibroblast-like synoviocytes (RA-FLSs) cells through mitochondrial/caspase-mediated signaling pathways (77). Migration inhibitory factor (MIF) is a pleiotropic inflammatory cytokine important in both innate and adaptive immune responses, and studies have demonstrated that elevated levels of MIF expression are observed in synovial tissues of RA patients compared to healthy individuals (78). Isopsoralen has been shown to ameliorate RA by targeting macrophage MIF, as evidenced by a significant decrease in serum production of IL-6, IL-1β, and cartilage oligomeric matrix protein (COMP) but an increase in IL-10 production in CIA mice (79).

SLE is a chronic autoimmune disease in which 75% to 80% of patients have skin manifestations, such as erythema of the cheeks, rashes, and skin ulcers (80). Double-negative (DN) T cells are defined by the lack of CD4 and CD8 and the ability to produce pro-inflammatory cytokines, such as IFN-γ, which have been implicated in the pathogenesis of SLE in humans and mice (81). Umbelliferone reduced DN T cells, plasma cells, IFN-γ+CD4+ T cells, and T follicular helper cells (CD3+TCRβ+CD4+CXCR5+PD1+) and increased the percentage of Treg cells in lupus nephritis MRL/lpr mice (82).

MS is a common clinical neuroimmune disease, which occurs in young and middle-aged people between the ages of 20 and 40, with more female patients than male patients, strong relapses, and a high disability rate (83). EAE is the classical animal model of MS. Different coumarins including daphnetin, plumbagin, umbelliferone, and osthole can treat EAE. Daphnetin attenuates EAE by up-regulating Th2 and Treg cells and inhibiting Th1 and Th17 cells, as evidenced by increased expression of anti-inflammatory cytokines and transcription factors (IL-4, IL-10, IL-33, GATA3, Foxp3), and decreased pro-inflammatory cytokines and transcription factors (IL-17, TNF-α, IFN-γ, STAT4, T-bet, STAT3, ROR-γt) production (84). In addition, daphnetin reduced pro-inflammatory cytokines, including IL-17, IFN-γ, IL-6, IL-12a, and IL-23a, in brain tissues of EAE mice. Heme oxygenase-1 (HO-1) is a typical antioxidant and anti-inflammatory factor. The study confirmed the ability of daphnetin to inhibit IL-1β, IL-6, and TNF-α production and significantly elevate HO-1 levels in lipopolysaccharide (LPS)-stimulated mouse BV2 microglial cells (85). A study looking at the effects of plumbagin on EAE found that plumbagin inhibited the differentiation, maturation, and function of human monocyte-derived DCs, as well as inhibited Th1 and Th17 cell polarization (decreasing the expression levels of IL-6, IL-1β, and IL-23), and promoted Th2 cell polarization (up-regulating the expression level of IL-4) (86). Umbelliferone attenuates clinical symptoms in EAE mice by inhibiting the activation of autoreactive T cells, suppressing Th1 cell polarization, and increasing the level of Foxp3+ regulatory T cells (87). The effects of osthole on EAE have also been reported. Osthole augments the therapeutic efficiency of neural stem cells and inhibits the reduction of nerve growth factor (NGF) and the elevation of IFN-γ in EAE mice (88, 89).

Psoriasis is primarily a refractory disease mediated by T-lymphocytes with a combination of genetic and environmental effects (90). Daphnetin treatment inhibits the proliferation and inflammatory response of human HaCaT keratinocytes and ameliorates imiquimod (IMQ)-induced psoriasis-like skin injury in mice and attenuates the IMQ-induced upregulation of inflammatory cytokines, including IL-6, IL-23A, and IL-17A (91). Treatment of psoriasis mice with an ointment containing osthole was found to reduce the secretion of TNF-α, IL-12, IL-17, and IL-23 in the skin of mice (92).

UC is a chronic non-specific inflammatory bowel disease, with lesions mainly located in the mucous membrane and submucosa. The main clinical manifestations include abdominal pain, diarrhea, and bloody stools (93). Treatment with decursin and decursinol inhibitthe production of IL-6, TNF-α, cyclooxygenase (COX)-2, hypoxia inducible factor (HIF)-1a, and prostaglandin E2 (PGE2) in colonic tissues of UC mice induced by dextran sulfate sodium (DSS) (94). Decursinol angelate ameliorates DSS-induced colitis by modulating Th17 cell responses, which is reflected in its ability to reduce the mRNA level of RORγt in Th17 cells and the expression of IL-17 in CD4+ T cells (95). In addition, plumbagin reduces the expression of circulating inflammatory monocytes (CD14+/CD16+) and cytokines (TNF-α and IFN-γ) in UC mice (96). Osthole treatment down-regulated the levels of pro-inflammatory Th1-associated cytokines (TNF-α) and Th17-associated cytokines (IL-17) and up-regulated the levels of anti-inflammatory Th2-associated cytokines (IL-4 and IL-10) (97). Another study confirmed that daphnetin can improve UC by regulating Treg/Th17 balance (98). In addition, the therapeutic effects of esculin, bergapten, esculetin and scoparone have been reported in UC (99–102).

Type 1 diabetes mellitus (T1DM) is an autoimmune disease in which pancreatic β cells are destroyed, resulting in an absolute lack of insulin (103). Umbelliferone increases the number of Foxp3+ regulatory T cells, thereby alleviating the severity of type 1 diabetes (104). In addition, imperatorin acts as a Takeda G-protein-coupled receptor 5 (TGR5) and G-protein-coupled receptor 119 (GPR119) agonist, inducing glucagon-like peptide (GLP-1) secretion and lowering blood glucose levels in type 1 diabetic rats through activation of TGR5 and GPR119 (105).

Chronic prostatitis (CP) is a common and intractable genitourinary chronic inflammatory disease in young and middle-aged men, characterized by slow onset, stubbornness, recurrent episodes, and intractability. The autoimmune response caused by the imbalance of CD4+ T cell differentiation was found to be an important etiological factor of CP. 4‐methylumbelliferone could reduce the severity of experimental autoimmune prostatitis (EAP) in experimental EAP mice by significantly decreasing the proportion of Th1 cells (106).

## 5 Coumarins targeting various signaling pathways

**Figure 3:**
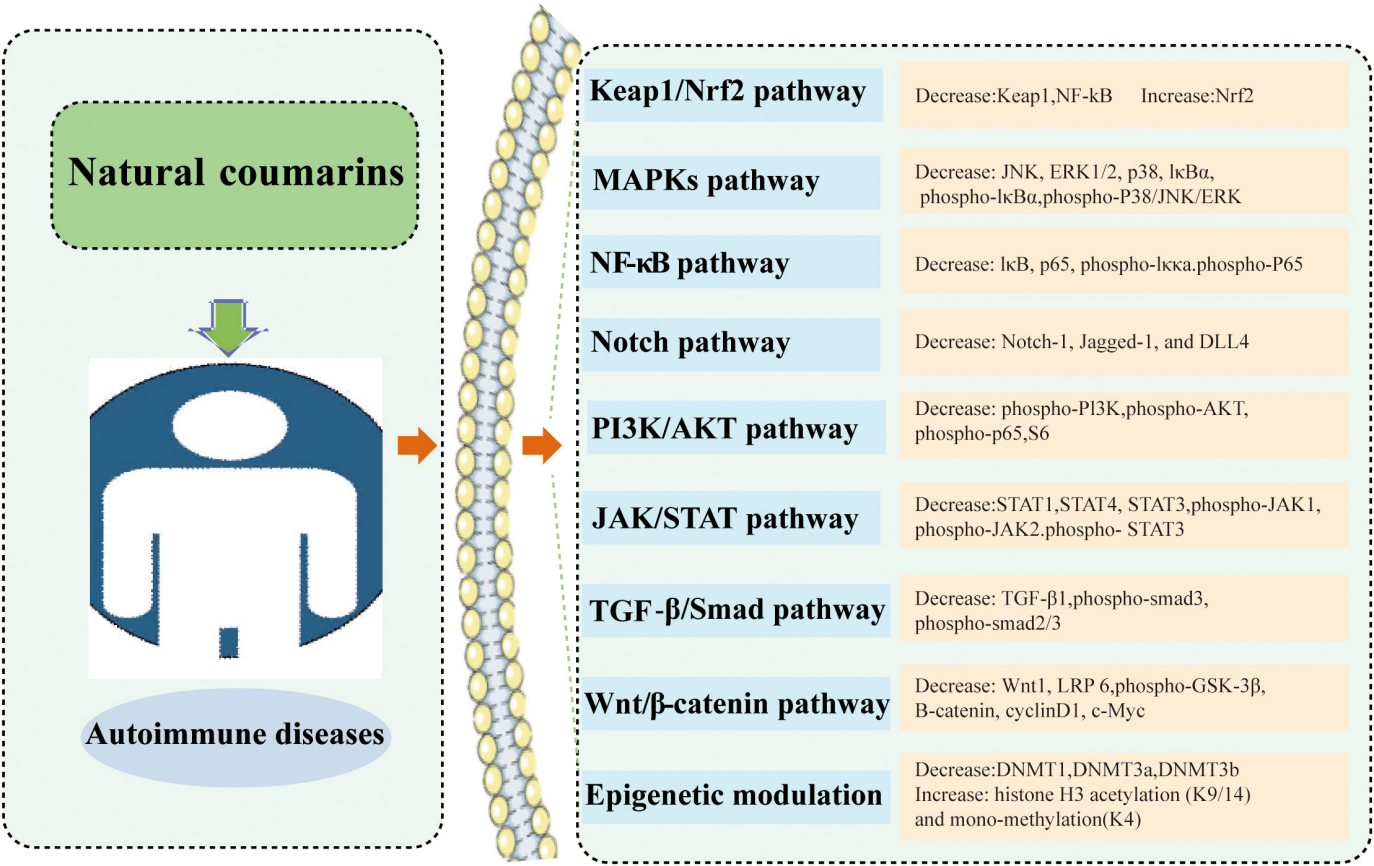
The main point of action of coumarins in autoimmune diseases.

Coumarin-like chemicals are notable for their anti-tumor properties. In recent years, accumulated studies have shown that coumarins exhibit promising immune regulatory effects in AIDs. Each type of coumarin targets different immune cells, thus triggering a large number of different intracellular signaling pathways, ultimately regulating the host’s immune response. The modulation of several signaling pathways leads to alterations in the expression of pro-inflammatory genes, which ultimately lead to an improvement in immune environment. To date, most of the mechanistic studies have been conducted in animal experiments. Many mechanistic studies have been conducted in animal and cell experiments. The action of coumarins in AIDs are summarized in **Figure 3** and **Table 1**.

**Table 1:** Coumarins targeting signaling pathways.

Signaling Pathways	Coumarins	Actions	References
MAPKs pathway	Osthole	Inhibiting the phosphorylation of IκB α and p38 pathway proteins alters pro-inflammatory cytokine production and expression	(111, 112)
	Fraxetin, imperatorin, plumbagin, daphnetin, esculetin	Suppressed the phosphorylation of ERK, JNK, AKT and p38 pathway proteins	(113–115, 117–119)
	4-methylumbelliferone	inhibiting ERK1/2 signaling and increasing the number of Foxp3^+^ T cells in an ERK1/2-dependent manner	(116)
	Osthole	Regulating the NLRP 3 inflammasome by activation of AMPK	(120)
TGF-β/Smad signaling pathway	Umbelliferone, esculetin	downregulated TGFβ1 and p-smad2/3 levels, while inhibiting nuclear translocation of NF-κB p65 and increasing Nrf 2 protein levels	(127, 128, 130)
	Esculetin	Reduced levels of TGF-β 1 and fibronectin	(129)
NF-κB signaling pathway	Umbelliferone, plumbagin, daphnetin, scopoletin, osthole, imperatorin, esculetin	Modulates NF-κB signaling pathway and reduces levels of pro-inflammatory cytokines, dendritic cells, and NLRP3 inflammasome.	(136–142, 147–149)
PI3K/AKT signaling pathway	Imperatorin	inhibits activation of the PI3K/AKT/NF-κB pathway	(153)
	Fraxetin	reduced phosphorylation of AKT and S6 levels and S6 protein levels	(113)
	Umbelliferone	inhibits AKT phosphorylation to prevent bone loss and suppresses osteoclastogenesis	(154)
Keap1/Nrf2 signaling pathway	Columbianadin	regulates the Keap1/Nrf2 signaling pathway and inhibits NF-κB activation	(157)
	Imperatorin	regulates the expression of Nrf-2, ARE and HO-1, thereby inhibiting pro-inflammatory cytokine secretion	(158)
	Esculetin	inhibiting complement activation and enhancing Nrf2 signaling pathway	(128)
	Umbelliferone	activating Nrf2 signal transduction	(159)
	Esculetin	decreases the expression of Keap1	(160)
Wnt/β-catenin signaling pathway	Umbelliferone	reduces the levels of pathway-associated proteins (Wnt1, LRP6, p-GSK-3β, β-catenin, cyclin D1, and c-Myc) and inhibits β-catenin nuclear translocation	(163, 164)
JAK/STAT signaling pathway	Plumbagin, daphnetin	inhibits the phosphorylation of STAT1, STAT3, and STAT4, as well as the upstream kinases JAK 1 and JAK 2	(170, 171)
	Colombianadin	inhibited the JAK1/STAT3 pathway to attenuate the inflammatory response and regulated the NF-κB pathway and Keap1/Nrf2 pathway	(157)
Epigenetic modulation	Umbelliferone	Activation of SIRT1 resulting in the inhibition of NF-κB, TLR4 and iNOS	(177)
	Osthole	Downregulates n6 -methyladenosine-modified TGM2 and attenuates the NF-κB signaling pathway	(179)
	Daphnetin	reduces gene expression of the methyltransferases DNMT1, DNMT3a and DNMT3b and demethylates the proapoptotic genes PDCD5, FasL, DR3 and p53	(178)
	Esculetin	restores histone H3 acetylation (K9/14) and mono-methylation (K4)	(129)
	Osthole	increases miR-1224-3P expression and decreases AGO1 expression and inhibits pro-inflammatory cytokine levels	(181)
Notch signaling pathway	Agrimonolide	decreasing the mRNA and protein levels of Notch-1, Jagged-1, and DLL4 and inhibiting the phosphorylation of JAK2 and STAT3	(186)

### 5.1 Mitogen-activated protein kinases (MAPKs) pathway

The family of MAPKs includes several subfamilies such as c-Jun n-terminal kinase (JNK), p38 MAPK, and extracellular signal-regulated kinases (ERK), which can regulate proliferation, differentiation, apoptosis, or survival, cellular activities such as inflammation and innate immunity (107, 108). There is connectivity and relative independence between different signaling pathways in the MAPK family. The c-Jun N-terminal kinase/stress-activated protein kinase (JNK/SAPK) and p38 kinase are activated by environmental stress and inflammatory signals, whereas extracellular signal-regulated kinase (ERK1/2/5) is mainly activated by growth factor receptors and some cytokine receptors. Once activated, MAPK will phosphorylate different proteins, acting as other kinase translation regulators and transcription factors, leading to cellular responses (109). Signals can be transmitted from the cell surface to the nucleus by activated MAPK to increase the expression of relevant inflammatory genes and promote the secretion of a variety of inflammatory factors, such as COX-2, PGE2, Monocyte chemoattractant protein-1 (MCP-1), IL-1β, IL-6, and TNF-α (110). Several studies have reported the inhibitory effects of coumarins on JNK, ERK1/2, and p38. These inhibitory effects lead to a reduction in the expression and release of pro-inflammatory mediators (IL-1β, IL-6, COX-2, MCP-1, e.g.). For example, osthole inhibited the expression of p38 MAPK, COX-2, inducible nitric oxide synthase (iNOS), and IκB α in LPS-induced RAW 264.7 cells and decreased the levels of NO, PGE2, TNF-α, and IL-6. On this basis, in DSS-induced UC mice, osthole decreased the expression of NF-κB p65 and p-IκB α in colonic tissues (111). Similarly, osthole significantly inhibited the phosphorylation of p38, which was induced by 2,4,6-Trinitrobenzenesulfonic acid (TNBS) in mice or by LPS in Raw264.7 cells, and strongly inhibited IL-1β, IL-6, COX-2, and MCP-1. Interestingly, the inhibition by protein kinase A (PKA) partially reversed the suppressive effects of osthole on p38 phosphorylation in LPS-stimulated cells (112). In endometriotic animal models and cells (End1/E6E7 and VK2/E6E7), fraxetin reduced endometriotic lesions by inhibiting P38/JNK/ERK phosphorylation, inducing apoptosis, and generating reactive oxygen species (ROS) (113). Imperatorin was able to attenuate symptoms associated with a mouse model of psoriasiform dermatitis by inhibiting the phosphorylation of ERK, JNK, and AKT. Meanwhile, the inhibitory effects of imperatorin on cell responses and signaling could be reversed by a PKA inhibitor, suggesting that cAMP/PKA is involved in the anti-inflammatory effects of imperatorin (114). In addition, inhibition of p38 activation by imperatorin has been reported (115). 4-methylumbelliferone (4-MU) inhibits ERK 1/2 signaling and increases the number of Fox P3+ T cells in an ERK1/2-dependent manner, thereby inhibiting hyaluronan synthesis to restore immune tolerance in autoimmune insulitis (116). Additionally, other coumarins, such as plumbagin, daphnetin, and esculetin have been shown to reduce inflammation by interfering with the MAPKs pathway (117–119).

It is noteworthy that the beneficial effects of coumarins may also be associated with increased signaling in the AMPKs pathway. For example, the anti-RA activity of osthole requires the involvement of AMPK phosphorylation activation. Osthole can regulate NLRP3 inflammasome by activating AMPK. This result was also reverse-validated by the experimental application of the AMPK inhibitor compound C, which blocked the activation of AMPK by osthole and also attenuated the positive effect of osthole on inflammasome activation, which was manifested as increased protein levels of NLRP3, CAS1, ASC and IL-1β (120).

### 5.2 Transforming growth factor beta/small mother against decapentaplegic (TGF-β/Smad) signaling pathway

The TGF-β/Smad signaling pathway is involved in many cellular processes (121). TGF-β is a multifunctional cytokine consisting of three isoforms, TGF-β1, TGF-β2, and TGF-β3, which is widely expressed in different types of cells and tissues, with TGF-β1 being the major isoform. TGF-β can negatively regulate immune cell proliferation, differentiation, and activation and plays an important role in suppressing immunity and inflammation (122, 123). Smad proteins, on the other hand, are signal transducers of intracellular TGF-β and mediate most of the functions of TGF-β (124). One of the mechanisms by which coumarins can modulate the immune response is through direct inhibition of the TGF-β/Smad signaling pathway. During diabetes, the expression of TGF-b is increased in the kidney, which leads to further deterioration of nephropathy (125). The circulating level of TGF-b1 is one of the important markers for predicting diabetes-related renal injury (126). Umbelliferone reduces Renal damage in type 1 diabetic rats by decreasing the levels of TGF-β1 in Renal tissue and circulation (127). In the MRL/lpr mouse model, esculetin significantly down-regulated the levels of TGFβ1 and p-smad3 in renal tissues, as well as significantly inhibited the nuclear translocation of NF-κB p65 and increased the level of Nrf2 protein in the nucleus, which had a significant therapeutic effect on murine lupus nephritis (128). In addition, esculetin treatment protects against the increase in expression of TGF-β1and fibronectin in type I diabetic rat kidney and hence shows efficacy in attenuating glomerulosclerosis (129). Another study reported that umbelliferone and esculetin could inhibit the activation of TGF-smad signal, which showed that they could down-regulate the secretion of fibronectin in HK2 cells stimulated by TGF-β1 and inhibit smad2/3 phosphorylation, thus playing a beneficial role in rats with type 1 diabetic nephropathy (130).

### 5.3 Nuclear factor kappa-light-chain-enhancer of activated B cells (NF-κB) signaling pathway

NF-κB is an important intracellular transcription factor that regulates the expression of a wide range of genes and plays a key regulatory role in a variety of biological processes including inflammatory response, cell proliferation, apoptosis, and cell infiltration (131). NF-κB is phosphorylated by IκB kinase upon cellular stimulation by chemical or mechanical signals and subsequently degraded via the ubiquitin-proteasome system. After IκB degradation, NF-κB dimers detached from IκB are activated by translation and enter the nucleus to participate in transcription (132–134). Activated NF-κB regulates the production of inflammatory factors such as TNF-α, COX-2, and PGE2 in the nucleus, and participates in and mediates a variety of immune responses and inflammatory reactions in the body (135). Different coumarins including umbelliferone, plumbagin, daphnetin, scopoletin, osthole, imperatorin, and esculetin all inhibit NF-κB pathways. In FLS of RA, umbelliferone and scopoletin counteract RA by binding to and inhibiting tyrosine kinases in RA-FLS and subsequently inhibiting NF-κB (136). Furthermore, umbelliferone ameliorates RA induced by complete Freund’s adjuvant by inhibiting the NF-κB signaling pathway in osteoclast differentiation (137). In a mouse model of EAE, scopoletin attenuates DCs activation through inhibition of the NF-κB signaling pathway and significantly reduces central nervous system (CNS) inflammation and demyelination in EAE mice (138). The inhibitory effect of daphnetin on NF-κB activation has been reported in various autoimmune disease models (psoriasis mice, EAE mice, NZB/WF1 SLE mice) (139–141). Plumbagin has been shown to reduce the levels of TNF-α, IL-6 and matrix metalloproteinases (MMPs) in RA mouse cells by inhibiting NF-κB activation, and its mechanism of action is related to the inhibition of IκB and NF-κB activation as well as the entry of p65 into the cell nucleus (142). Furthermore, in patients, IL-1β plays a pathogenic role in the evolution of IgA nephropathy (143), and serum levels of IL 18 are elevated in IgA nephropathy patients (144). Mature IL-1β and IL-18 are produced by active caspase-1 from NLRP3 inflammasome from their respective precursors pro- IL-1β and pro IL-18 (145, 146). In a mouse model of progressive IgA nephropathy, osthole blocked the activation of NF-kB and NLRP3 inflammasome, thereby improving renal function and blocking progressive renal lesions (147). IL-1β A study observing the effects of esculetin on skin inflammation in psoriasis mice found that esculetin inhibited the activation of the NF-κB signaling pathway, including inhibiting the phosphorylation of IKKα and P65 in psoriatic skin (148). In addition, the inhibitory effect of imperatorin on NF-κB has been reported in mice with UC (149).

### 5.4 Phosphatidylinositol 3-kinase/protein kinase B (PI3K/Akt) signaling pathway

The PI3K/AKT pathway is an important signaling pathway in the body, consisting of two protein kinases, PI3K and AKT, which are involved in the phosphorylation of NF-κB p65 and nuclear translocation, and contribute to the production of inflammatory mediators (150, 151). Overall, the PI3K/AKT pathway activates the signaling pathway upon stimulation of the corresponding upstream signals, which in turn directs the downstream signaling substances as well as the cytosolic nucleus to make the corresponding response, and further regulates the phenomena of cell autophagy, apoptosis, and inflammation release, which ultimately affects the development of diseases (152). It is hypothesized that the beneficial effects of coumarins may be related to the inhibition of signaling pathways in the PI3K/AKT pathway. It was confirmed that imperatorin significantly inhibited the activation of the PI3K/AKT/NF-κB pathway by inhibiting the phosphorylation levels of PI3K, AKT, and p65 in the ectopic endometrium tissue, thereby significantly inhibiting the growth and ameliorate the histopathological features of ectopic endometrium in experimental endometriosis rats (153). Another study reported that fraxetin significantly reduced phosphorylation of AKT and S6 levels and S6 protein levels in End1/E6E7 and VK2/E6E7 cells (endometriotic epithelial cell lines) (113). Furthermore, umbelliferone prevents LPS-induced bone loss and inhibits RANKL-induced osteoclastogenesis by inhibiting AKT phosphorylation (154).

### 5.5 Kelch-like-ech-associated protein 1-nuclear factor E2-related factor 2 (Keap1/Nrf2) signaling pathway

Kelch-like-ech-associated protein 1 (Keap1)-nuclear factor E2-related factor 2 (Nrf2) pathway is closely related to oxidative stress and inflammation in various organs and systems of the body, and is considered as the therapeutic target of many organ protection. Nrf2 is the main regulator of cell antioxidant response, and its activity is precisely regulated by the negative regulatory protein Keap1. The antioxidant effect of Nrf2 was inhibited by the interaction with Keap1 (155, 156). The imbalance of Keap1/Nrf2 transcription activity is related to the pathogenesis of many diseases. Keap1/Nrf2 axis has become the most important regulator of intracellular homeostasis and plays an important role in the occurrence and development of many chronic diseases. Some studies have reported the regulatory effects of coumarin on Nrf2 and Keap1. For example, in the collagen-induced RA mouse model, columbianadin can play an anti-RA role by regulating inflammation and oxidative stress, and its mechanism includes inhibiting the expression of Keap1 at mRNA and protein levels, increasing the expression of Nrf2 mRNA in CIA mice, regulating Keap1/Nrf2 signaling pathway in CIA mice, and inhibiting the activation of NF-κB (157). In addition, imperatorin has been proven to interfere with the expression of Nrf2 in the colon of rats with UC induced by TNBS, and inhibit the secretion of TNF-α and IL-6 by regulating the expressions of Nrf-2, ARE and HO-1, thus alleviating the symptoms of UC (158). Umbelliferone can also alleviate UC induced by DSS by inhibiting inflammation, which is related to activating Nrf2 signal transduction (159). Other studies have reported that esculetin can treat lupus nephritis in mice by inhibiting complement activation and enhancing Nrf2 signaling pathway (128). In addition, the inhibition of esculetin on Keap1 activation has also been reported. In a study, it was reported that esculetin can reduce the expression of Keap1 in aorta of hyperinsulinemia combined with T1DM rats, and has a protective effect on vascular function (160).

### 5.6 Wngless-type/beta-catenin (Wnt/β-catenin) pathway

The Wnt/β-catenin signaling pathway, also known as the Canonical Wnt signaling pathway, is a conserved signaling axis (161). The Wnt/β-catenin pathway consists of four segments: the extracellular signaling, membrane segment, cytoplasmic segment, and nuclear segment. Extracellular signaling is mainly mediated by Wnt proteins, among which are Wnt3a, Wnt1, and Wnt5a. The cytosolic fragment mainly contains the Wnt receptor Frizzled and low-density lipoprotein receptor-related protein (LRP5/6). The cytoplasmic fraction mainly consisted of β-catenin, Dishevelled (DVL), glycogen synthase kinase-3β (GSK-3β), AXIN, adenomatous polyposis coli (APC) protein, and casein kinase-1 (CK-1). Nuclear segments mainly include β-catenin translocated to the nucleus, T-cell factor/lymphoid enhancer factor family (TCF/LEF), and β-catenin downstream target genes such as MMPs and c-Myc (162). Coumarins can alter the Wnt/β-catenin pathway along multiple steps in the signaling cascade. Umbelliferone reduces Wnt1 protein levels, activates GSK-3β kinase by blocking GSK-3β (Ser9) phosphorylation, and reduces the protein level and nuclear translocation of β-catenin (163). Furthermore, in FLS from RA rats, Umbelliferone could reduce the activation of the Wnt/β-catenin pathway by restoring GSK-3β activity, reducing the levels of pathway-associated proteins (e.g., Wnt1, LRP6, p-GSK-3β (Ser 9), β-catenin, cyclin D1, and c-Myc), and inhibiting β-catenin nuclear translocation (164).

### 5.7 Janus kinase/signal transducer and activator of transcription (JAK/STAT) pathway

The JAK-STAT pathway is a signaling pathway from the cell membrane to the nucleus and is critical in apoptosis, proliferation and differentiation, body immune function, and inflammatory response (165, 166). The JAK-STAT pathway consists of JAK-associated receptors, JAK, and STAT (167). Among them, the Janus kinase family is a class of non-receptor-type protein tyrosine kinases including JAK1, JAK2, JAK3, and tyrosine kinase 2 (TYK2) (168). STAT is a class of cytosolic proteins, located downstream of JAK, including STAT1, STAT2, STAT3, STAT4, STAT5a, STAT5b, and STAT6 (169). These molecules contribute to the inflammatory process and, by inference, their inhibition represents a therapeutic target for the reduction of inflammation. Thus, the mechanism by which coumarins can exert immunomodulatory effects may be the inhibition of these molecules. Plumbagin significantly inhibited the phosphorylation of STAT1, STAT4, and STAT3, as well as the upstream kinases JAK1 and JAK2, resulting in a reduction in the number of CD4+ T-lymphocytes and pro-inflammatory cytokines in mice with experimental autoimmune encephalomyelitis, which ameliorated the locomotor dysfunction and body weight loss of mice (170). Similar results were observed in LPS-induced Caco-2 cells, where daphnetin inhibited the phosphorylation of JAK2 and STAT3 proteins (171). In addition, in mice models of CIA, colombianadin was able to exert anti-RA effects by modulating immune and inflammatory responses, and its mechanism of action included decreasing the phosphorylation levels of JAK1 and STAT3 in the ankle joints of mice with CIA as well as the STAT3 mRNA expression, suggesting that colombianadin attenuates inflammatory responses by inhibiting the JAK1/STAT3 pathway. It is worth mentioning that columbianadin also inhibited the protein expression of P65, P50, and phosphorylated IκBα in the ankle joints of mice, inhibited the expression of Keap1 at the mRNA and protein levels, and increased the expression of Nrf2 at the mRNA level in CIA mice (157).

### 5.8 Epigenetic modulation

More and more studies show that epigenetic modification can regulate the inflammatory response and immune response through DNA, histone, transcriptional, and post-transcriptional levels (44, 172). Indeed, a series of studies have reported the existence of coumarin-induced epigenetic modifications leading to gene activation or silencing in the absence of changes in DNA sequence (173–176). A novel point of coumarins in cellular control is their ability to modulate modular epigenetic mechanisms such as DNA methylation, histone modifications, and posttranscriptional regulation of microRNAs, thereby regulating immune cell activation and differentiation. Among various coumarins, umbelliferone has been shown to be a strong activator of Silent information regulator 1 (SIRT1), leading to down-regulation of gene and protein expression of TLR4, NF-κB, and iNOS signaling factors, as well as decreasing the levels of TNF-α, IL-6, MPO, and VCAM-1 in the colon, resulting in a potent anti-inflammatory effect in acetic acid-induced UC rats (177). It is reported that esculetin can attenuate the decrease in histone H3 acetylation (K9/14) and mono-methylation (K4) in the kidney of rats with type I diabetic nephropathy induced by streptozotocin (STZ) (129). In addition, daphnetin had a demethylating effect on the proapoptotic genes PDCD5, FasL, DR3, and p53 in CIA rat synovial cells, and decreased the gene expression of the methyltransferases DNMT1, DNMT3a, and DNMT3b (178). In a study, osthole downregulated n6-methyladenosine-modified TGM2 to exert its additive effect with methotrexate and suppress the proliferation, migration, and invasion of RA-FLSs by attenuating NF-κB signaling pathway, resulting in the suppression of RA progression (179).

MicroRNAs are small and non-coding regulatory RNAs that can regulate the translocation and/or degradation of messenger RNAs (180). The regulatory effect of coumarin on microRNAs was also reported. It is reported that osthole can increase the expression of microRNA-1224-3p (miR-1224-3p) and decrease the expression of AGO1 in HUM-iCell-s010 RA cells, and decrease the levels of IL-6 and IL-1β in these cells. This discovery suggests that osthole may have the potential to treat RA by regulating the expression of miR-1224- 3 P and AGO 1 and reducing the level of proinflammatory cytokines (181).

### 5.9 Other pathways

The Notch signaling pathway is a conserved and important mechanism for maintaining immune homeostasis by regulating cell differentiation and modulating inflammation (182). In mammals, the pathway includes ligands (e.g., Jagged1, Jagged2, Delta1, Delta3, and Delta4), Notch receptors (Notch1-4), and downstream signaling components (183, 184). Aberrant activation of the Notch signaling pathway disrupts Th17/Treg cell homeostasis (185). Agrimonolide was able to correct the imbalance of Th17/Treg cells by significantly decreasing the mRNA and protein levels of Notch-1, Jagged-1, and DLL4, as well as inhibiting the phosphorylation of JAK2 and STAT3, which effectively attenuated the symptoms of weight loss and hematochezia, decreased the expression of inflammatory cytokines, and repaired intestinal mucosal barrier in UC mice (186). The Hedgehog (HH) pathway is critical for embryonic development and homeostatic maintenance of many adult tissues and organs. It is also associated with certain functions of the innate and adaptive immune system (187). HH, including sonic hedgehog (SHH), Indian hedgehog (IHH), and desert hedgehog (DHH) (188). FLSs are the main effector cells responsible for synovitis and joint destruction in RA. Studies have shown that the SHH signaling pathway is involved in the aberrant activation of RA-FLSs, and inhibition of the SHH pathway reduces the proliferation and migration of RA-FLSs (189). Therefore, the Hedgehog signaling pathway may be one of the pathways of coumarins for the treatment of autoimmune diseases, and it also provides a reference for the further development and utilization of coumarins.

## 6 Conclusions

Therapeutic agents available for the treatment of AIDs are limited, and there are certain shortcomings, such as dosage, route of administration, and bioavailability. Some therapeutic drugs have varying degrees of side effects. Given these limitations, we reviewed various coumarins that shown promising efficacy in AIDs such as T1DM, UC, SLE, RA, MS. Coumarin can regulate inflammatory cytokines such as IL-4, IL-6, IL-10, IL-17, IL-23, TNF-α, and IFN-γ, as well as related signaling pathways in immune cells, including JAK-STAT, Wnt/β-catenin, PI3K-AKT, TGF-β/Smad, MAPKs, Keap1/Nrf2, Notch and NF-κB pathways. The review provides new evidence for the discovery of effective and safe new drugs for AIDs.
